# Patterns of radiotherapy practice for biliary tract cancer in Japan: results of the Japanese radiation oncology study group (JROSG) survey

**DOI:** 10.1186/1748-717X-8-76

**Published:** 2013-04-01

**Authors:** Fumiaki Isohashi, Kazuhiko Ogawa, Hirobumi Oikawa, Hiroshi Onishi, Nobue Uchida, Toshiya Maebayashi, Naoto Kanesaka, Tetsuro Tamamoto, Hirofumi Asakura, Takashi Kosugi, Takashi Uno, Yoshinori Ito, Katsuyuki Karasawa, Makoto Takayama, Yoshihiko Manabe, Hideya Yamazaki, Mitsuhiro Takemoto, Yasuo Yoshioka, Kenji Nemoto, Yasumasa Nishimura

**Affiliations:** 1 Department of Radiation Oncology, Osaka University Graduate School of Medicine, 2-2 (D-10) Yamadaoka, Suita, Osaka, 565-0871, Japan; 2 Department of Radiology, Iwate Medical University, 19-1 Uchimaru, Morioka, Iwate, 020-8505, Japan; 3 Department of Radiology, University of Yamanashi, 1110, Shimogato Chuo, Yamanashi, 409-3898, Japan; 4 Department of Radiation Oncology, Shimane University, 1060 Nishikawatsu-cho, Matsue-shi, Shimane, 690-8504, Japan; 5 Present affiliation: Department of Radiation Oncology, Tottori Prefectural Central Hospital, 730 Etsu, Tottori-shi, Tottori, 680-0901, Japan; 6 Department of Radiology, Nihon University School of Medicine, 30-1, Ohyaguchi-Kamimachi, Itabashi-ku, Tokyo, 173-8610, Japan; 7 Department of Radiology, Tokyo Medical University, 6-1-1, Shinjuku, Shinjuju-ku, Tokyo, 160-8402, Japan; 8 Department of Radiation Oncology, Nara Medical University School of Medicine, 840 Shijo-cho, Kashihara, Nara, 634-8521, Japan; 9 Division of Radiation Oncology, Shizuoka Cancer Center, 1007 Shimonagakubo, Nagaizumi Town, Shizuoka, 411-8777, Japan; 10 Department of Radiology, Hamamatsu University School of Medicine, 1-20-1 Handayama, Higashi-ku, Hamamatsu city, Shizuoka, 431-3192, Japan; 11 Department of Radiology, Chiba University Graduate School of Medicine, 1-8-1 Inohana, Chiba, 260-8677, Japan; 12 Department of Radiation Oncology, National Cancer Center Hospital, 5-1-1 Tsukiji, Chuo-ku, Tokyo, 104-0045, Japan; 13 Department of Radiation Oncology, Tokyo Metropolitan Komagome Hospital, 18-22, Honkomagome 3chome, Bunkyo-ku, Tokyo, 113-8677, Japan; 14 Department of Radiology, Kyorin University School of Medicine, 6-20-2 Shinkawa, Mitaka-shi, Tokyo, 181-8611, Japan; 15 Department of Radiology, Nagoya City University Graduate School of Medical Sciences, Kawasumi, Mizuho-cho, Mizuho-ku Nagoya, Aichi, 467-8601, Japan; 16 Department of Radiology, Kyoto Prefectural University of Medicine, Kajii-cho, Kawaramachi-Hirokoji, Kamigyo-ku, Kyoto, 602-8566, Japan; 17 Department of Radiology, Okayama University, 2-5-1 Shikata-cho, Kita-ku, Okayama-shi, Okayama, 700-8558, Japan; 18 Department of Radiation Oncology, Yamagata University, 2-2-2 Iida-Nishi, Yamagata-shi, Yamagata, 990-9585, Japan; 19 Department of Radiation Oncology, Kinki University Faculty of Medicine, 377-2, Ohno-Higashi, Osaka-Sayama, Osaka, 589-8511, Japan

**Keywords:** Biliary tract cancer, Radiotherapy, Chemotherapy, Adjuvant, Palliative

## Abstract

**Background:**

The patterns of radiotherapy (RT) practice for biliary tract cancer (BTC) in Japan are not clearly established.

**Methods:**

A questionnaire-based national survey of RT used for BTC treatment between 2000 and 2011 was conducted by the Japanese Radiation Oncology Study Group. Detailed information was collected for 555 patients from 31 radiation oncology institutions.

**Results:**

The median age of the patients was 69 years old (range, 33–90) and 81% had a good performance status (0–1). Regarding RT treatment, 78% of the patients were treated with external beam RT (EBRT) alone, 17% received intraluminal brachytherapy, and 5% were treated with intraoperative RT. There was no significant difference in the choice of treatment modality among the BTC subsites. Many patients with EBRT were treated with a total dose of 50 or 50.4 Gy (~40%) and only 13% received a total dose ≥60 Gy, even though most institutions (90%) were using CT-based treatment planning. The treatment field consisted of the primary tumor (bed) only in 75% of the patients. Chemotherapy was used for 260 patients (47%) and was most often administered during RT (64%, 167/260), followed by after RT (63%, 163/260). Gemcitabine was the most frequently used drug for chemotherapy.

**Conclusions:**

This study established the general patterns of RT practice for BTC in Japan. Further surveys and comparisons with results from other countries are needed for development and optimization of RT for patients with BTC in Japan.

## Background

Biliary tract cancer (BTC) is a rare disease that is curable by surgery in fewer than 10% of all cases. Prognosis depends in part on the anatomic location of the tumor, which affects its resectability. Total resection is possible for 25% to 30% of lesions originating in the distal bile duct, a rate that is clearly better than that for lesions in more proximal sites. However, the rate of relapse is as high as 60-75%, even if clear resection (R0 resection) is possible [1]. In many patients with a tumor that cannot be completely removed by surgery, other treatments such as radiotherapy (RT) or stenting procedures may maintain adequate biliary drainage and improve survival. Optimal management is therefore essential for both postoperative and unresectable BTC.

In Japan, there were an estimated 20,734 new cases of BTC in 2007, with more than a 3-fold increase over the last three decades [2], while RT has become much more common because new methods and technology for treatment planning are now available. For these reasons, optimal management of RT for BTC has become a major concern in Japan. For the study presented here, the Japanese Radiation Oncology Study Group (JROSG) conducted a nationwide questionnaire-based survey on BTC. The questionnaire elicited detailed information regarding patient characteristics, treatment characteristics, and outcomes of treatment. The primary goal of this study was to determine the patterns of RT practice for BTC in order to provide assistance with development of future randomized clinical trials. Therefore, factors influencing the treatment outcome are analyzed elsewhere (Yoshioka et al.: Factors influencing survival outcome in radiotherapy for biliary tract cancer, submitted). To the best of our knowledge, this is the first report to establish how RT is used nationally to treat BTC in Japan.

## Methods

The JROSG conducted a nationwide survey of RT used for BTC treatment between 2000 and 2011 using a questionnaire requesting detailed information on patients and treatment characteristics. Patients were included if they met the following criteria: diagnosis of BTC without evidence of distant metastasis; treatment with RT between 2000 and 2011; no diagnosis of any other malignancy; and no previous RT. Diagnosis of BTC without pathologic verification was based on radiographic findings from contrast-enhanced computed tomography (CT), ultrasonography, endoscopic ultrasonography, and endoscopic retrograde/magnetic resonance cholangiopancreatography.

Of the 71 radiation oncology centers in Japan belonging to the JROSG, 31 (40%) agreed to participate in the survey. The other centers did not participate mostly because too few BTC patients had been treated with RT at the center in the study period. Each participating center provided a database of patients with BTC treated with RT between 2000 and 2011. The study was performed according to guidelines approved by the institutional review board of each institution whenever necessary.

The Mann–Whitney *U* test and Student’s *t*-test were used to investigate relationships between variables. A p value of < .05 or a 95% confidence interval not including 1 was considered to be statistically significant. All statistical tests were 2-sided.

## Results

### Data collection

**Figure 1:**
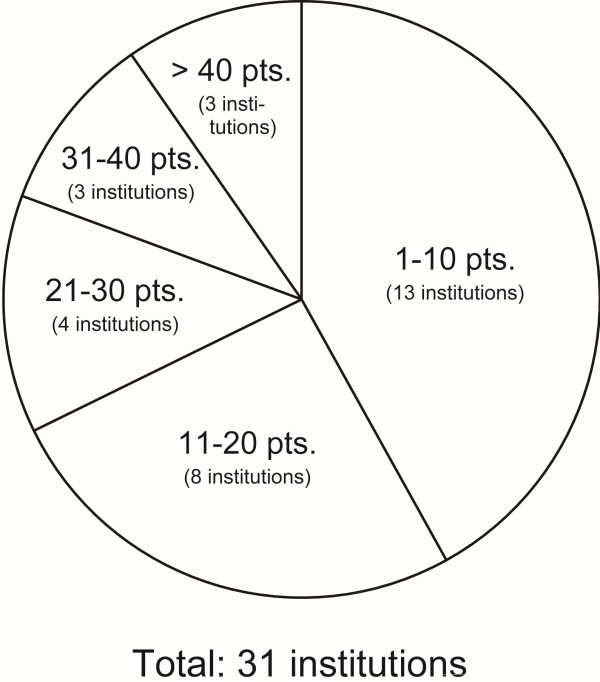
**Distribution of institutions by number of patients treated during 2000–2011.** The number of patients varied considerably among institutions.

Detailed information was collected for 555 patients from 31 institutions with a median of 15 patients per institution (range: 1–56 patients). The distribution of the number of institutions based on the number of patients treated between 2000 and 2011 is shown in Figure 1. This indicates considerable variation among institutions in the number of patients treated during the 11-year period: ≤10 patients were treated at 13 institutions (42%), while over 30 patients were treated at only 6 institutions (19%).

### Patient and disease characteristics

The background characteristics of all 555 patients are listed in Table 1. The median age was 69 years old (range, 33–90 years old) and 48% of the patients were ≥70 years old. Pre-therapeutic evaluations were performed by ultrasonography, CT, and magnetic resonance cholangiography in 81%, 93%, and 58% of the patients, respectively. Regarding the primary site, ~50% of BTC lesions arose in the perihilar regions of the extrahepatic bile duct, with distal regions of the extrahepatic bile duct being the second most common site (26%). Among all patients, >80% had an Eastern Cooperative Oncology Group performance status of 0–1, ~30% had a drinking or smoking habit, 52% had an unresectable tumor at diagnosis, and 53% had clinical stage T3-4 disease at diagnosis.

**Table 1:** Patient and disease characteristics (n = 555)

**Characteristic**	**Patients (%)**
**Age (median, 69 y)**	
< 70 y	288 (51.9)
≥ 70 y	267 (48.1)
**Gender**	
Female	183 (33.0)
Male	372 (67.0)
**Pathologic type, verified**	
Yes, adenocarcinoma	417 (75.1)
Yes, other	5 (0.9)
No	133 (24.0)
**Ultrasonography (before RT)**	
Yes	451 (81.3)
No	21 (3.8)
Unknown	83 (14.9)
**CT (before RT)**	
Yes	515 (92.8)
No	5 (0.9)
Unknown	35 (6.3)
**MRCP (before RT)**	
Yes	324 (58.4)
No	152 (27.4)
Unknown	79 (14.2)
**PTCD**	
Yes	242 (43.6)
No	151 (27.2)
Unknown	162 (29.2)
**Primary site**	
Intrahepatic bile duct	71 (12.8)
Gallbladder	42 (7.6)
Extrahepatic bile duct	439 (79.1)
Perihilar	278 (50.0)
Distal	144 (25.9)
Unknown	17 (3.1)
Ampulla of Vater	3 (0.5)
**Maximal tumor size (Median, 4.0 cm)**	
< 4.0 cm	195 (35.1)
≥ 4.0 cm	198 (35.7)
Unknown	162 (29.2)
**Tumor emboli**	
Yes	32 (5.8)
No	292 (52.6)
Unknown	231 (41.6)
**ECOG performance status**	
0	223 (40.2)
1	226 (40.7)
2	77 (13.8)
3	17 (3.1)
4	1 (0.2)
Unknown	11 (2.0)
**Jaundice**	
Yes	355 (64.0)
No or unknown	200 (36.0)
**CA19-9 (U/mL)**	
< 37	102 (18.4)
37-1,000	253 (45.6)
≥ 1,000	81 (14.6)
Unknown	119 (21.4)
**CEA (ng/ml)**	
< 5	300 (54.1)
5-10	63 (11.3)
≥ 10	49 (8.8)
Unknown	143 (25.8)
**Alcohol consumption**	
Yes	193 (34.8)
No	223 (40.2)
Unknown	139 (25.0)
**Smoking**	
Yes	175 (31.5)
No	239 (43.1)
Unknown	141 (25.4)
**Diabetes mellitus**	
Yes	75 (13.5)
No	383 (69.0)
Unknown	97 (17.5)
**Clinical T stage**	
TX	11 (2.0)
T1	41 (7.4)
T2	147 (26.4)
T3	183 (33.0)
T4	112 (20.2)
Unknown	61 (11.0)
**Clinical N stage**	
N0	310 (55.9)
N1	165 (29.7)
Unknown	80 (14.4)
**Clinical stage**	
I	96 (17.3)
II	202 (36.4)
III	146 (26.3)
IV	25 (4.5)
Unknown	86 (15.5)
**Resectable at diagnosis**	
Yes	254 (45.8)
No	288 (51.9)
Unknown	13 (2.3)
**Investigational protocol**	
Yes	0 (0)
No	555 (100)

Abbreviations: *RT* Radiotherapy; *CT* Computed tomography; *MRCP* Magnetic retrograde cholangiopancreatography; *PTCD* Percutaneous transhepatic cholangiodrainage; *ECOG* Eastern cooperative oncology group; *CEA* Carcinoembryonic antigen; *CA19-9* Carbohydrate antigen 19–9.

### Characteristics of surgical procedures

Primary surgery before RT was performed in 242 patients (44%). Curative surgery was performed in 235 patients, but only 63 (26% of those who underwent surgery) had complete (R0) resection. R1 resection (microscopic positive margins) and R2 resection (macroscopic residual tumor) were performed in 142 (59%) and 37 (15%) patients, respectively. Note that surgeries included non-curative (R2) and curative-intent (R0 or R1) resections, because our cohort was based on a RT database. Lymph node dissection was performed on 173 patients (71%) and a positive node was identified pathologically in 85 patients (35%).

### Radiation treatment characteristics

The most common treatment modality was external beam radiotherapy (EBRT) alone (78% of the patients), followed by intraluminal brachytherapy (ILBT) with or without EBRT (17%) and intraoperative RT (IORT) with or without EBRT (5%). Chemotherapy before, during, or after RT was used for 260 patients (47%).

**Figure 2:**
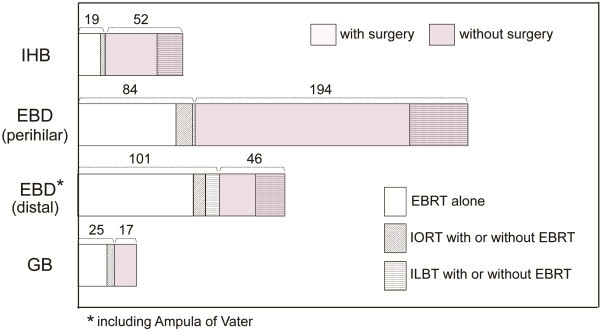
**Patterns of radiation practice or choice of treatment modality by BTC subsites.** There was no significant difference in the choice of treatment modality among the BTC subsites.

The patterns of RT practice or choice of treatment modality according to the BTC subsites are shown in Figure 2. Because the subsites of 17 patients were unknown, the patterns for 538 patients were analyzed. The rate of primary surgery varied according to the tumor subsite: primary surgery was performed for only 30% of tumors that originated in proximal regions (intrahepatic and perihilar), but for 67% of those that originated in more distal lesions (distal and gallbladder) (*p* < .05). However, there was no significant difference in the choice of treatment modality among the BTC subsites.

Table 2 shows the treatment modality choices according to purpose of RT, which was divided into four groups: RT after curative resection (R0-1) (n = 183), RT after non-curative resection (R2) (n = 33), curative RT for inoperable cases (n = 235), and palliative RT for inoperable cases (n = 78). The purpose of RT for inoperative cases (curative or palliative) was chosen by radiotherapists who answered the questionnaire. Twenty-six patients with IORT were excluded from this analysis based on a comparison of doses among the variables because strong bias was suspected when a parameter such as IORT was used, which involved a very large dose at one time. Over 90% of the patients who underwent surgery received EBRT alone. For the patients who did not undergo surgery, there was a tendency for ILBT with EBRT to be used for a curative purpose more often than for a palliative purpose, but the difference was not statistically significant (25% vs. 15%, *p* = .08). To compare the combined dose of ILBT and EBRT with a single modality dose (ILBT alone or EBRT alone), the total dose (ILBT + EBRT) was calculated as the biologically equivalent dose in 2-Gy fractions (EQD_2_) using the linear quadratic model. The value used for assessing effects on tumors was α/β = 10 Gy. The median EQD_2_ for EBRT alone, ILBT alone and EBRT with ILBT was 50 Gy_α/β10_, 36 Gy_α/β10_, and 60Gy_α/β10_, respectively, while that for ILBT with EBRT was significantly greater than EBRT alone or ILBT alone (*p* = .001). In terms of treatment purpose, however, there were no significant differences in the median EQD_2_ among the groups (50Gy_α/β10_ for all variables).

**Table 2:** Choices of treatment modality according to purpose of RT (n = 529)

	**Purpose of RT**		**Treatment modality (%)**			**median EQD**_**2**_**(range) Gy**_**α/β10**_
		**Actual patients**	**EBRT alone**	**ILBT alone**	**ILBT + EBRT**	
**Surgery+**	Curative intent (R0-1)	183	170 (92.9)	8 (4.4)	5 (2.7)	50 (6–90)
	Non-curative intent (R2)	33	31 (93.9)	1 (3.0)	1 (3.0)	50 (4–74)
**Surgery-**	Curative	235	177 (75.3)	0 (0)	58 (24.7)	50 (9–68)
	Palliative	78	55 (70.5)	11 (14.1)	12 (15.4)	50 (39–74)
	median EQD_2_ (range) Gy_α/β10_		50 (4–90)	36 (14–44)	60 (33–82)	

Abbreviations: *RT* Radiotherapy; *EBRT* External beam radiotherapy; *ILBT* Intraluminal brachytherapy; *EQD2* The biologically equivalent dose in 2-gray fractions.

### EBRT characteristics

The characteristics of the 521 patients who received EBRT are shown in Table 3. The median duration from surgery to EBRT was 34 days (range, 9–88 days). EBRT was administered with ≥3 portals to 69% of the patients, at ≥10-megavolt beam energy for >90%, and at 1.8 Gy or 2.0 Gy per fraction; and with a total dose of ≥40 Gy for ~90%. CT-based treatment planning and conformal RT were used for 90% and 64%, respectively, of patients treated with EBRT, but only two of these patients received intensity-modulated RT (IMRT).

**Table 3:** EBRT characteristics (n = 521)

**Characteristic**	**Patients (%)**
**EBRT Radiation portals**	
2 portals	162 (31.1)
≥ 3 portals	359 (68.9)
**EBRT beam energy (MV)**	
< 10	24 (4.6)
≥ 10	491 (94.2)
Unknown	6 (1.2)
**EBRT dose/fraction (Gy)**	
< 1.8	7 (1.3)
1.8	131 (25.1)
2	352 (67.6)
> 2.0	31 (6.0)
**EBRT total radiation dose (Gy)**	
< 40	69 (13.2)
40 - < 50	129 (24.8)
50/50.4	206 (39.5)
> 50.4 - < 60	52 (10.0)
≥ 60	65 (12.5)
**Radiation field**	
primary only	388 (74.5)
primary plus regional LN	119 (22.8)
LN only	5 (1.0)
Unknown	9 (1.7)
**CT-based treatment planning**	
Yes	468 (89.8)
No	53 (10.2)
**Conformal therapy**	
Yes	333 (63.9)
No	75 (14.4)
Unknown	113 (21.7)
**IMRT**	2 (0.38)

Abbreviations: *EBRT* External beam radiotherapy; *MV* Megavolt; *Gy* Gray; *LN* Lymph node; *CT* Computed tomography; *IMRT* Intensity-modulated radiotherapy.

A summary of the EBRT field based on performance of surgery and nodal status is shown in Table 4. The treatment field consisted of the primary tumor only in 388 (75%) of 521 patients and the primary tumor plus regional lymph nodes in 119 (23%). Patients who underwent surgery received RT for the primary tumor (bed) plus regional lymph nodes more frequently than patients who did not undergo surgery (29% vs. 19%, *p* < .01). Additionally, among the patients who underwent surgery, RT for the primary tumor (bed) plus regional lymph nodes of those with clinically positive nodes was more frequently performed than in patients with clinically negative nodes (31% vs. 12%, *p* < .01). However, patients with pathologically positive nodes tended to receive RT for the primary tumor (bed) plus regional lymph nodes more frequently than patients with pathologically negative nodes, but the difference was not statistically significant (37% vs. 27%, *p* = .16). Among patients who did not undergo surgery, RT for the primary tumor and regional lymph nodes of those with clinically positive nodes was more frequently performed compared to patients with clinically negative nodes (56% vs. 6%, *p* < .01). However, some patients with clinically positive nodes also underwent EBRT for the primary tumor only (43%).

**Table 4:** EBRT field according to performance of surgery and N stage (n = 521)

		**Radiation field (%)**		
**Group**	**Patients (n)**	**Primary**	**Primary plus LN**	**Others**
**Surgery +**				
Total	219	151 (68.9)	63 (28.8)	5 (2.2)
pN0	75	54 (72.0)	20 (26.7)	1 (1.3)
pN1	78	49 (62.8)	29 (37.2)	0 (0)
Unknown	66	48 (72.7)	14 (21.2)	4 (6.1)
cN0	111	95 (85.6)	13 (11.7)	3 (2.7)
cN1	65	43 (66.2)	20 (30.8)	2 (3.0)
Unknown	43	13 (30.2)	30 (69.8)	0 (0)
**Surgery -**				
Total	302	237 (78.5)	56 (18.5)	9 (3.0)
cN0	189	171 (90.5)	11 (5.8)	7 (3.7)
cN1	79	34 (43.0)	44 (55.7)	1 (1.3)
Unknown	34	32 (94.2)	1 (2.9)	1 (2.9)
**Total**	521	388 (74.5)	119 (22.8)	14 (2.7)

Abbreviation: *EBRT* External beam radiotherapy; *LN* Lymph node.

**Figure 3:**
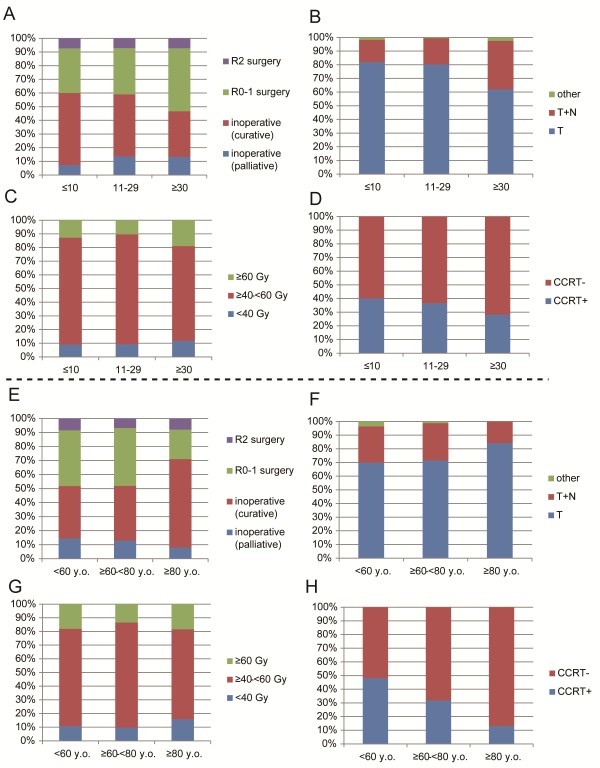
**Practice patterns of EBRT according to caseload of institutions or patient age.** Three categories were formed based on the number of patients treated at each institution (≤10, 11–29 and ≥30 patients) (**A-D**) or age (<60, ≥60- < 80, and ≥80 years old) (**E-H**) and evaluated based on treatment intent (**A, E**), EBRT field size (**B, F**), EBRT total dose (**C, G**), and concurrent chemotherapy (**D, H**).

Analyses of practice patterns of EBRT were performed according to caseload of institutions (Figure 3a-d) and patient age (Figure 3e-h). Caseloads were divided into three categories based on the number of patients treated within the study period at each institution (≤10, 11–29, and ≥30 patients). In institutions with ≥30 patients, the rates of postoperative RT (compared to inoperable cases) (Figure 3A), EBRT for the field of the tumor (bed) plus regional LN (compared to tumor only) (Figure 3B), and patients receiving ≥60 Gy (Figure 3C) were significantly higher than those in institutions with <30 patients. Age was also divided into three categories (<60, ≥60- < 80, and ≥80 years old). The use of CCRT was significantly higher in patients <60 years old compared to those ≥60- <80 years old, and in those ≥60- < 80 years old compared to those ≥80 years old (Figure 3H).

### ILBT and IORT characteristics

A total of 96 patients (17%) received ILBT at 13 institutions (42%). The characteristics of these cases are listed in Table 5. All 96 patients were treated with ILBT using an iridium-192 source and at 5 or 6 Gy per fraction in 55% of cases and with a total dose of ≥15 Gy in 85%, 76 (79%) of whom received ILBT with EBRT at a median EBRT dose of 40 Gy (range, 20–60 Gy). The most common prescription point was 10 mm from the source (75%).

**Table 5:** Intraluminal brachytherapy (n = 96)

**Characteristic**	**Patients (n)**
**Source**	
Ir-192	96 (100)
**ILBT single dose/fraction (Gy)**	
< 5	16 (16.7)
5	33 (34.4)
6	20 (20.8)
> 6	27 (28.1)
**Total dose (Gy)**	
< 15	14 (14.6)
15 - 25	41 (42.7)
≥ 25	41 (42.7)
**Prescription point (from the source)**	
5 mm	4 (4.2)
7 mm	4 (4.2)
10 mm	72 (75.0)
12 mm	14 (14.6)
Unknown	2 (2.1)
**With EBRT** (Median EQD_2,_ 60.4 Gy)	76 (79.2)
**Without EBRT** (Median EQD_2,_ 35.8 Gy)	20 (20.8)

Abbreviations: *Ir* Iridium; *Gy* Gray; *EBRT* External beam radiotherapy; *EQD*_*2*_ The biologically equivalent dose in 2-gray fractions.

IORT was used for only 26 patients (5%) at four institutions (13%, 4/31), 12 (2%) of whom received IORT with EBRT and 14 (3%) received IORT alone. The median dose for IORT was 25 Gy (range, 20–30 Gy), with a median beam energy of 12 mega-electron volts (range, 4–25 mega-electron volts).

### Chemotherapy

Chemotherapy was used for 260 patients (46%), including 167 concurrently with RT (78 concurrently alone; 7 pre-RT and concurrently; 67 concurrently and post-RT; and 15 pre-RT, concurrently, and post-RT), 4 pre- and post-RT, 12 pre-RT alone, and 77 post-RT alone. The drugs and timing of chemotherapy for these patients are listed in Table 6. Chemotherapy was most often given during RT (64%, 167/260) followed by after RT (63%, 163/260), while the most frequently used drug for chemotherapy was gemcitabine (47%) followed by 5-FU (37%). TS-1 and UFT were especially frequently used after RT.

**Table 6:** Drugs used and timing of chemotherapy (n = 260)

**Variable**	**Actual patients (%)**	**Chemotherapy timing (%)**		
		**Before RT**	**During RT**	**After RT**
**Actual patients (n)**	260	38	167	163
**Drugs**				
GEM	122 (46.9)	24 (63.2)	72 (43.1)	78 (47.9)
5-FU	97 (37.3)	9 (23.7)	74 (44.3)	43 (26.4)
Cisplatin	40 (15.4)	9 (23.7)	22 (13.2)	15 (9.2)
TS-1	45 (17.3)	6 (15.8)	5 (3.0)	42 (25.8)
UFT	34 (13.1)	3 (7.9)	12 (7.2)	24 (14.8)
Other	9(3.4)	3 (7.9)	4 (2.4)	2 (1.2)

Abbreviations: *RT* Radiotherapy; *GEM* Gemcitabine; *5-FU* 5-Fluorouracil; *TS-1* Tegafur-gimeracil-oteracil potassium; *UFT* Tegafur-uracil.

**Figure 4:**
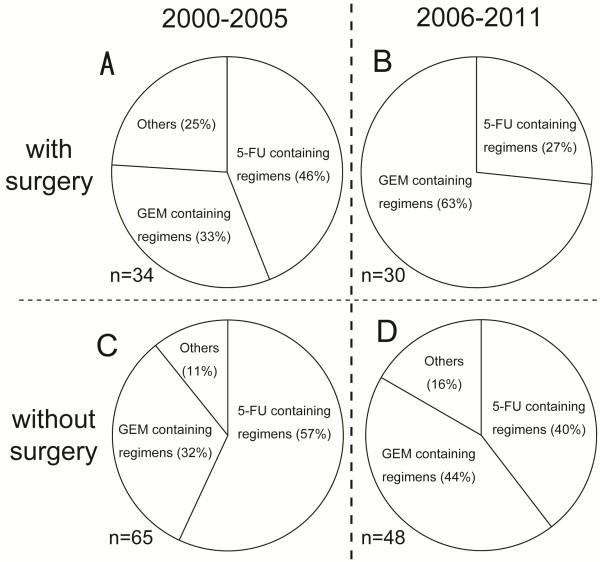
**Changes in chemotherapy regime combined with radiotherapy during 2000–2011 based on prior therapy.** The patients were divided into four groups according to performance of surgery and the timing during the study period. **A**: Surgery, 2000–2005 (n = 24); **B**: Surgery, 2006–2011 (n = 30); **C**: No surgery, 2000–2005 (n = 65); **D**: No surgery, 2006–2011 (n = 48).

The 167 patients who received chemotherapy during RT (concurrent chemoradiation (CCRT)) were analyzed further because this method has been shown to be efficacious for treatment of patients with BTC with or without surgery. The patients were divided into four groups according to performance of surgery and timing during the study period: Group A, surgery, 2000–2005 (n = 24); Group B, surgery, 2006–2011 (n = 30); Group C, no surgery, 2000–2005 (n = 65); and Group D, no surgery, 2006–2011 (n = 48). There was a significant difference in the use of gemcitabine-containing regimens between Groups A and B and between Groups C and D (Figure 4). This suggests a trend away from the use of 5-FU towards a more frequent use of gemcitabine concurrently with RT for patients with BTC treated with or without surgery.

## Discussion

RT for BTC can be classified into adjuvant therapy after surgery or therapy for inoperable cases. While no randomized control trial has been conducted, a meta-analysis revealed that patients with extrahepatic cholangiocarcinoma treated with adjuvant RT show a significantly lower mortality rate than patients treated with surgery alone [3]. Data in the Surveillance Epidemiology and End Result database also suggest that palliative RT prolongs survival in patients with extrahepatic cholangiocarcinoma [4]. In these reports, the outcomes of the treatment were reported in detail, but detailed information on RT use has not been provided and there are few reports on patterns of RT practice. We therefore decided to evaluate the practice of RT for BTC at Japanese radiation oncology centers, with the goal of assisting with development of randomized clinical trials. JROSG has conducted similar surveys and successfully determined the general patterns of RT practice for several other cancers in Japan [5,6]. Of the 31 responding institutions, 43% treated fewer than 10 patients over the period covered by the survey. Surprisingly, none of the patients were treated with an investigational protocol, clearly indicating a need for a prospective multicenter study to determine a standard therapeutic approach.

The results of the study showed that CT-based treatment planning was used for approximately 90% of the patients. Previous nationwide surveys of the structural characteristics of radiation oncology in Japan found that only 329 (45%) of 726 facilities in 2003 and 407 (57%) of 712 facilities in 2005 used CT-based treatment planning [7,8]. These results suggest that three-dimensional conformal RT planning became mainstream during the survey period or that patients with BTC received RT more frequently in facilities with advanced equipment.

We examined the variations in RT use (modality, total dose, or RT fields) according to the purpose of RT or BTC subsites. Some analyses have suggested that there is a dose–response relationship for treatment of BTC and have stressed the importance of dose escalation [9,10]. However, many patients with EBRT included in this survey were treated with a total dose of 50 or 50.4 Gy (~40%) and only 13% of the patients received a total dose ≥60 Gy. These data indicate that use of sufficient doses for EBRT for tumors in the hepatic hilum and liver regions was severely restricted by technical difficulties with the delivery of high doses to these regions while sparing surrounding organs, including the liver, duodenum, stomach, and spinal cord, even though most institutions used CT-based treatment planning. Recently, IMRT has emerged as a sophisticated technique for treatment of tumors, including BTC, in areas at risk of recurrence, while sparing adjacent normal tissue from high-dose irradiation [11]. However, only two patients were treated with IMRT for EBRT during the survey period.

ILBT can also be used for dose escalation in a region at risk [9,12] since it has the advantage of allowing delivery of a sufficient dosage to a target focus while reducing the effect of irradiation on surrounding tissues. Theoretically, a combination of ILBT and EBRT can enhance the beneficial effects of RT, with fewer adverse effects than those incurred with EBRT alone. In fact, ILBT with EBRT entailed a significantly higher EQD_2_ dose than EBRT alone in our study cohort. While 42% of the institutions performed ILBT, only 14% of all patients received ILBT combined with EBRT, indicating that this treatment modality was used only in selected cases because the effect of ILBT is limited to the area surrounding the lumen of the biliary tract and improvement in local control can therefore be expected only for small tumors [9].

The optimal radiation field for BTC remains to be defined. The majority of relapses after resection with curative intent occur at the primary tumor site [13], which suggests that it may be reasonable to limit RT to the primary tumor (bed). Only 23% of the patients included in this survey received radiation to the tumor (bed) as well as the regional lymph nodes, regardless of the lymph node status. Although limiting the radiation field to the tumor (bed) has tended to become prevalent in Japan, the definition of clinical target volume included regional lymph nodes as well as the tumor (bed) in a recent meta-analysis of 14 selected papers with detailed information on adjuvant RT after surgery [3], as well as in many reports on unresectable BTC published since 2000 [14-17]. Collectively, these findings indicate that the radiation field for BTC is not yet standardized due to the lack of a large randomized control trial and that additional studies investigating the optimal radiation field should be conducted.

The study presented here showed that chemotherapy is frequently administered in combination with RT (47% of all patients). Chemotherapy was most often administered during RT, followed by after RT. Several trials have examined the efficacy of adjuvant chemoradiation after surgery [18] or of chemoradiation for unresectable cases [19]. The National Comprehensive Cancer Network (NCCN) reported that most CCRT for BTC involved the use of 5-FU, and that CCRT with gemcitabine is not recommended due to the limited experience with and potential toxicity of this treatment. However, the use of CCRT combined with gemcitabine-containing regimens increased in Japan during the period covered by the current survey, which suggests that additional studies should be undertaken to establish the optimal sequencing of RT and chemotherapy with drugs such as gemcitabine. For chemotherapy for advanced BTC, the recent randomized control phase III ABC-02 study showed that a combination of gemcitabine and cisplatin improved overall and progression-free survival by 30% over gemcitabine alone [20]. Based on these results, the combination of gemcitabine and cisplatin can now be considered to be the standard of care as first-line chemotherapy for patients with advanced or metastatic BTC. In Japan, however, oral anticancer drugs such as TS-1 or UFT also tend to be used as adjuvant chemotherapy after RT, and only two patients in the current study were treated with a combination of gemcitabine and cisplatin after RT.

## Conclusions

Patients with BTC should continue to be enrolled in prospective studies of RT with radiosensitizing agents or of RT with dose escalation methods using techniques such as IMRT. Further surveys and comparisons with results from other countries are needed for development and optimization of RT for patients with BTC in Japan.

## Consent

Written informed consent was obtained from the patient for publication of this report and any accompanying images.

## Abbreviations

RT: Radiotherapy; BTC: Biliary tract cancer; EBRT: External beam radiotherapy; JROSG: The Japanese radiation oncology study group; CT: Computed tomography; ILBT: Intraluminal brachytherapy; IORT: Intraoperative radiotherapy; EQD2: The biologically equivalent dose in 2-Gy fractions; IMRT: Intensity-modulated radiotherapy; CCRT: Concurrent chemoradiotherapy; NCCN: National comprehensive cancer network.

## Competing interests

The authors made no disclosures and not receive specific funding.

## Authors’ contributions

KO coordinated the entire study. Patient data acquisition was done by FI, HO, HO, NU, TM, NK, TT, HA, TK, TU, YI, KK, MT, YM, HY, MT, KN, and YN. Data analysis was done by FI, KO, and YY. The manuscript was prepared by FI. Corrections and/or improvements were suggested by KO and YY. Revisions were done by HO, HO, NU, TM, NK, TT, HA, TK, TU, YI, KK, MT, YM, HY, MT, KN, and YN. All authors read and approved the final manuscript.
